# Response to Long-Term NaHCO_3_-Derived Alkalinity in Model *Lotus japonicus* Ecotypes Gifu B-129 and Miyakojima MG-20: Transcriptomic Profiling and Physiological Characterization

**DOI:** 10.1371/journal.pone.0097106

**Published:** 2014-05-16

**Authors:** María Florencia Babuin, María Paula Campestre, Rubén Rocco, Cesar D. Bordenave, Francisco J. Escaray, Cristian Antonelli, Pablo Calzadilla, Andrés Gárriz, Eva Serna, Pedro Carrasco, Oscar A. Ruiz, Ana B. Menendez

**Affiliations:** 1 Instituto de Investigaciones Biotecnológicas-Instituto Tecnológico de Chascomús/Universidad Nacional de General San Martín-Consejo Nacional de Investigaciones Científicas y Técnicas (IIB-INTECH/UNSAM-CONICET), Chascomús, Argentina; 2 Unidad Central de Investigación en Medicina-INCLIVA, Universitat de Valencia, Valencia, Spain; 3 Departamento de Bioquímica y Biología Vegetal-Universitat de Valencia, Valencia, Spain; 4 Departamento de Biodiversidad y Biología Experimental, Facultad de Ciencias Exactas y Naturales, Universidad de Buenos Aires, Buenos Aires, Argentina; Key Laboratory of Horticultural Plant Biology (MOE), China

## Abstract

The current knowledge regarding transcriptomic changes induced by alkalinity on plants is scarce and limited to studies where plants were subjected to the alkaline salt for periods not longer than 48 h, so there is no information available regarding the regulation of genes involved in the generation of a new homeostatic cellular condition after long-term alkaline stress. *Lotus japonicus* is a model legume broadly used to study many important physiological processes including biotic interactions and biotic and abiotic stresses. In the present study, we characterized phenotipically the response to alkaline stress of the most widely used *L. japonicus* ecotypes, Gifu B-129 and MG-20, and analyzed global transcriptome of plants subjected to 10 mM NaHCO_3_ during 21 days, by using the Affymetrix *Lotus japonicus* GeneChip®. Plant growth assessment, gas exchange parameters, chlorophyll a fluorescence transient (OJIP) analysis and metal accumulation supported the notion that MG-20 plants displayed a higher tolerance level to alkaline stress than Gifu B-129. Overall, 407 and 459 probe sets were regulated in MG-20 and Gifu B-129, respectively. The number of probe sets differentially expressed in roots was higher than that of shoots, regardless the ecotype. Gifu B-129 and MG-20 also differed in their regulation of genes that could play important roles in the generation of a new Fe/Zn homeostatic cellular condition, synthesis of plant compounds involved in stress response, protein-degradation, damage repair and root senescence, as well as in glycolysis, gluconeogenesis and TCA. In addition, there were differences between both ecotypes in the expression patterns of putative transcription factors that could determine distinct arrangements of flavonoid and isoflavonoid compounds. Our results provided a set of selected, differentially expressed genes deserving further investigation and suggested that the *L. japonicus* ecotypes could constitute a useful model to search for common and distinct tolerance mechanisms to long-term alkaline stress response in plants.

## Introduction

Plant salt stress represents a large economic problem worldwide, which has been the subject of countless studies. However, given that the agricultural surface on alkaline soils is higher than that on saline ones, it is not salinity *per se* but the generation of alkaline or mixed salt-alkaline stresses the main source of constraint for farming [1]. Soils owing its alkalinity to high Na_2_CO_3_ and NaHCO_3_ contents are extended throughout practically all climatic regions. In these soils, the stressor factors for plant growth are high pH, high exchangeable sodium percent, poor fertility, dispersed physical properties, and very low water infiltration capacity. Besides, alkalinity affects the solubility of essential micronutrients such as iron and zinc [2], [3].

Global gene expression using cDNA-microarrays has allowed the identification of groups and networks of genes that respond to diverse stresses such as cold, drought and salinity in rice [4], or drought in barley [5]. The same technique has also allowed the identification of genes that respond to alkalinity in *Glycine soja* [6], [7], *Glycine max* [8], *Leymus chinensis* [9], *Puccinellia tenuiora* [10], [11] and *Limonium bicolor* [12].

The synthesis of polypeptides in response to variations in the environment was suggested to be biphasic: first proceeds the synthesis of “early” proteins, which are implicated in the stress perception and signalling, and this is followed by the synthesis of ‘late” proteins, involved in the recovery of a normal cellular metabolism [13]. In the above mentioned microarray studies on alkalinity, plants were subjected to alkaline stress for periods not longer than 48 h, so the outcoming information from those studies would be mainly implicated in the perception and transduction of the stress signal. In contrast, no long-term study on plants grown under alkalinity has been so far undertaken and hence, there is no information available regarding the regulation of genes involved in the generation of a new homeostatic cellular condition.

Legumes (Fabaceae) are the major source of plant proteins for human consumption and livestock feed, as well as key components of natural and agricultural ecosystems. Within this legume family is *Lotus,* a genus comprising several species acknowledged by their elevated adaptability to divers soil constrains. *Lotus* species are used as an alternative forage in South America and Australia, and cover crop for dunes revegetation and reclamation of heavy metal-contaminated or burned soils in Europe [14]. In addition, the species *L. japonicus* has become a model legume [15] due to its characteristic genome features, and the development of a variety of resources for functional genomics, which has helped in the advance of legume research during the last years [16].

The *L. japonicus* genome sequencing project has led to the development of an Affymetrix GeneChip® handling more than 52000 *Lotus* probe sets, which are representative of most of the known and predicted open reading frames (ORFs), including biotic and abiotic stress-responsive genes [16]. Microarray profiling using the *Lotus* Genechip allowed the identification of 912 probe sets that were differentially expressed under the acclimatization of *L. japonicus* to salt stress [17], and has been useful to study the role of plastidic glutamine synthetase (GS2) in proline biosynthesis and drought stress responses in this species [18]. On other hand, it has been suggested that natural variation between cultivars or accessions, when coupled with high-throughput sequencing and quantitative phenotyping for improved stress tolerance, can pinpoint candidate genes for future study [19].

The aim of the present study was two-fold: first, to phenotipically characterize the response to alkaline stress of the most widely used *L. japonicus* ecotypes [20], Gifu B-129 and Miyakojima MG-20; and to identify genes regulated by long-term alkaline stress, with view to increase the current knowledge on plant response to soil alkalinity. With this purpose, we analyzed global transcriptome of these two experimental accessions under alkaline stress (Gifu B-129 sequence data, gene information in *L. japonicus* and mapping information available at http://www.kazusa.or.jp/lotus).

## Materials and Methods

### Plant Material and Growth Conditions

Seeds from *L. japonicus* ecotypes MG-20 and Gifu B-129 were scarified with sulfuric acid (100%) 3 min, washed ten times with sterile distilled water and sown in Petri dishes containing water-agar (0.8%). Plates were incubated for 7 days in a growth chamber, with a 16/8 h photoperiod at 24°C/21°C ±2°C (day/night) and 55/65±5% relative humidity. Light intensity (250 µmol m^−2^ s^−1^) was provided by Grolux fluorescent lamps (F 40W). One seedling was transferred to each cylindrical pot (5.8×9×20 cm; volume = 0.53 dm^3^) containing washed sand mix (50% fine/50% coarse sand; pH 7.0; E.C. = 0.05 mS cm^−1^) and irrigated with 0.5×Hoagland’s nutrient solution [21] containing 3 mM KNO_3_; 2 mM Ca(NO_3_)_2_.4H_2_O; 1 mM MgSO_4_.7H_2_O; 0.5 mM NH_4_H_2_PO_4_; 0.5 NaFeO_8_EDTA.2H_2_O; and 0.5 mM of each of the following micronutrients: MnCl_2_.4H2O, H_3_BO_3_, CuSO_4_.5H_2_O, ZnSO_4_.7H_2_O, and Na_2_MoO_4_.2H_2_O. A drip irrigation system (9001 Digital Watering Timer Weekly Program, ELGO®, www.elgo.co.il; flow rate = 6.25 ml/h) was used according to Paz *et al*. [22].

### Experimental Design

Experiments followed a completely randomized design. Two-way ANOVA analysis was performed (ecotype×treatment). Two ecotypes were evaluated: *L. japonicus* MG-20 and *L. japonicus* Gifu B-129, under two treatments: control without salt addition and alkalinity. Measurements of growth, gas exchange, OJIP, and iron and zinc contents were performed on 12 plants ( = 12 biological replicates). For microarray analysis, four replicates per treatment were used, each replicate consisted of 24 pooled plants.

### Alkaline Treatment

Alkaline stress treatment was imposed during 21 days by adding NaHCO_3_ 10 mM to the 0.5×Hoagland’s solution and was started when plants were at the two full developed leaves stage. Control treatment consisted of plants irrigated with 0.5×Hoagland’s solution without NaHCO_3_. The pH and E.C. of irrigation solutions were monitored every 3 days with a combined pH meter/conductimeter (HI 255, Hanna Instrument) and maintained at pH/E.C. (mS cm^−1^) 6.2/1.2 and 8.2/1.9, for control and alkaline treatments, respectively. For microarray analysis, plants were harvested, divided into shoots and roots, frozen in liquid N_2_ and stored at −80°C until they were processed for total RNA extraction. For dry matter, plants were dried at 60°C until constant weight.

### Gas Exchange Measurements

The gas exchange parameters, transpiration rate (E), stomatal conductance (Gs), net photosynthesis rate at light saturation (Pn), were measured in one apical and one basal leaf per plant at light saturation (1200 µmol photons m^−2^ s^−1^ illumination, LED light) using a portable photosynthesis system (TPS-2 Portable Photosynthesis System, MA, USA).

### Chlorophyll Content Determination

Leaves were harvested and stored at −80°C until use. For pigments extraction, 40 mg of plant material grounded in liquid nitrogen was shaken in 100% acetone (4°C, overnight). The extract was cold centrifuged and the supernatant removed. Measurements were made at wavelength 663 nm (chlorophyll a) and 647 nm (chlorophyll b) in a spectrophotometer (Perkin Elmer Lambda 25 UV/VIS spectrometer), and pigments concentration calculated according to Lichtenthaler et al. [23].

### Iron and Zinc Determinations

To analyze total Fe and Zn concentrations, *Lotus* roots, leaves and stems were collected, carefully washed with deionized water and deposited in glass vials. For dry matter destruction, dry ashing was performed on 100 mg of material at 550°C for 8 h. Samples were digested with 0,5 ml of HNO_3_ 65% and completed to 3,5 ml final volume with deionized water. An Analyst 100 atomic absorption spectrophotometer (Perkin Elmer), absorption mode was used.

Active Fe was extracted and assayed according to Chen and collaborators [24] with some modifications. Roots, stems and leaves were harvested, frozen in liquid N_2_ and stored at −80°C until analysis. The different organs were cut into small pieces with a scissor. Tissue samples (100 mg) of each organ were shaken in 1,2 mL of 80 mM 2,2′-dipyridyl-HCl (pH 3.0) in 10% methanol in the dark (4°C; 24 h). Extracts were passed through a 0.45 µm syringe filter and 1 mL of the filtrate was assayed at 522 nm using a spectrophotometer (Zeltec ZL-5000 UV/VIS Spectrometer, Argentina). Active Fe values were calculated from a standard curve using Fe atomic absorption standard solution.

### Chlorophyll Fluorescence Fast-transient Analysis

One day before harvest, non-invasive O-J-I-P analysis was performed in the first full developed leaf by chlorophyll fluorometry with a portable chlorophyll fluorometer (PocketPea, Hansatech Instrument, UK). Leaves were predarkened 20 min before analysis and then exposed during 3 s to 3500 µmol photons m^−2^ s^−1^ (637 nm peak wavelength). The maximum quantum yield of primary photochemistry (*F*V/*F*M) was calculated. In addition, the contribution to photosynthesis regulation by the three functional steps namely absorption of light energy (ABS), trapping of excitation energy (TR) and conversion of excitation energy to electron transport (ET) was expressed through the multi-parametric expression performance index (PI_ABS_; [25]).

### RNA Isolation and Transcriptomic Analysis

Total RNA was extracted from frozen shoots and roots using a Plant Spectrum Total RNA Kit (Sigma), according to the manufacturer’s instructions. RNA quality was checked on agarose gel electrophoresis. Total RNA (300 ng/sample) was labeled using the Affymetrix GeneChip® cDNA Synthesis and Amplification Kit protocol, and hybridized to the arrays as described by the manufacturer. Arrays were scanned on an Affymetrix GeneChip® Scanner 3000 7G. A GeneChip® Operating Software supplied by Affymetrix was used to perform the gene expression analysis. Data (.CEL files) were analyzed and statistically filtered using Robin software [26]. Input files were normalized with the RMA algorithm and statistically significant genes were identified using mixed model analysis of variance with a false discovery rate (fdr correction) of *P<*0.05. Fold-change values <+2 and >−2 were removed. Functional classification of significant genes was analyzed using MapMan software [27], [28] after converting fold change values to log_2_ in Excel files. The data (.CEL files) presented in this publication have been deposited in the ArrayExpress database (http://www.ebi.ac.uk/arrayexpress/) and are accessible through the accession number E-MTAB-2418.

### Quantitative RT-PCR

To validate microarray values by quantitative PCR, 2.5 µl from a tenfold dilution of the cDNA stock was further diluted to 15 µl with the primer mix (300 nM final concentration), 7.5 µl of FastStart Universal SYBR® Green Master (Rox) and the required amount of double distilled water. Primers used in these reactions are listed in Table 1. Reactions were performed in a Mx3005P qPCR System with the help of the MxPro qPCR Software 4.0 (Stratagene, La Jolla, CA, U.S.A.). Relative quantification was carried out by the comparative cycle threshold method with the *1αEF* (ID: AY633710) as an endogenous control [29].

**Table 1:** Primers used for quantitative real-time PCR.

Probeset ID	Forward primer	Reverse primer
ljwgs_124992.1_at	AAGTTGTCATCCAAGTTG	GTAGTAGTTCATATTCACCAT
ljwgs_011581.2_at	AAGTTGTCATCCAAGTTG	GTAGTAGTTCATATTCACCAT
ljwgs_086126.1_at	GAGCACTTGAACATTGAA	TCCACTAACATCCTTGAG
chr5.cm0456.15_at	CGGATTACTACCTTGACA	TGATTGAAGAAGCAAAGTG
Ljwgs_012445.1_at	ATGCTGTAACCATCTGAAT	GCCAATAATCACTGAATG
chr6.cm0437.7_at	GGGTCCAAAGAGAAAGTT	AGTCACATCAAGCACATAA
cm0528.2_at	CTCGTCAAACAACTTCAC	CAATGGCACAAATCCTAAA
ljwgs_038566.1_at	CTTCATCAGCAACAATCAT	AAGCAATACCAGTTCCAA
ljwgs_049882.1_at	TGATGACTCCTCAGAACTT	CCTATGATTACAGAATGAACAAC
ljwgs_021886.2_at	TGAGCTTGTGAAGGTTGG	AACAGGGAGTTGACAAATCT
chr1.cm0378.1_at	AAGATGGAGAGGGATATGG	GTCTTGTTCTCACGCTTT
chr3.cm0279.2_at	TGGAGGTCATAGTAGTATCT	GAGGACTCACTTCTTCAT
ljwgs_063085.1_x_at	ATACAACTACAGCGTCAT	GCAATCAATTTGGACTCA
chr5.cm0019.23_at	TTTAACCCTCATAGTCCT	ATTGCTAGTGAAGACATC
chr1.cm0433.18_at	ACTTGGTGTTAAGGAGATT	TTGTCTGAATGGAGTTGA
ljwgs_147904.1.1_at	TGGTGAGTGGAGGAAGTG	TAGCATTGACAGAGATGAAGAG
ljwgs_055792.1_at	CCAATCTTCAACATTCCAA	CTATCTTACAAGGCTCAGT
chr1.cm0800.52_at	CTCTTGGTCTTCTTCTCAATACA	ATAGCAACGAATGGCATCA
*1αEF* (Housekeeping gene)	TGACAAGCGTGTGATCGAGAGG	GATACCTCTTTCACGCTCAGCCTT

For comparative purposes, relative gene expression in control plants was defined as 1. The INFOSTAT [30] software tool was used to calculate the relative expression ratios on the basis of group means for target gene transcripts versus the reference gene transcript [31].

## Results and Discussion

### Phenotypic Characterization. Plant Growth, OJIP Analysis, and Fe and Zn Contents

**Figure 1:**
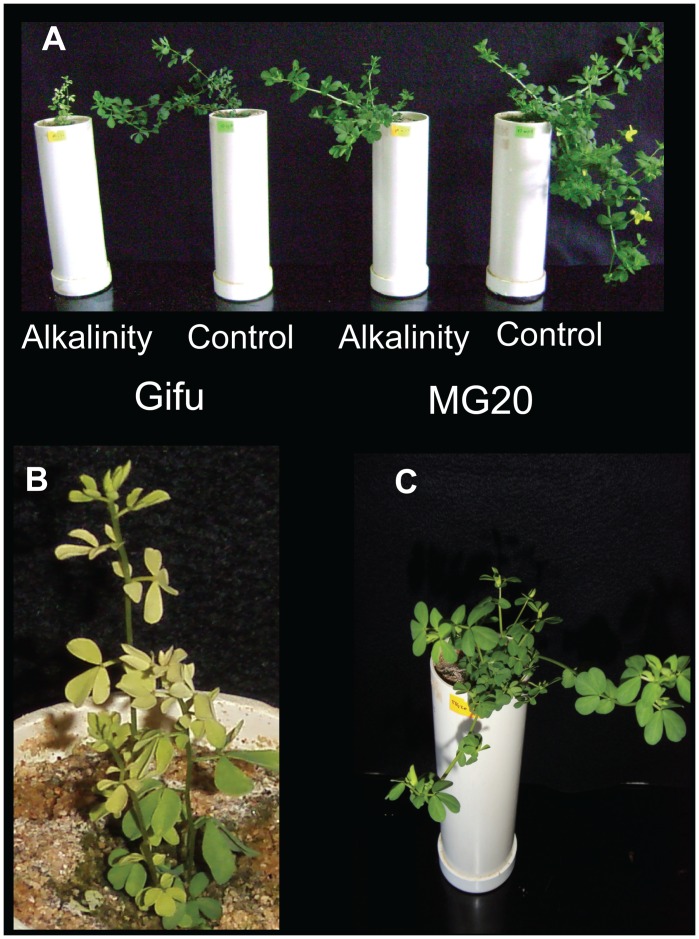
Plant growth response to alkalinization of the two *L. japonicus* ecotypes Gifu B-129 and MG-20. Plants of both ecotypes grown under alkalinity and control treatments (A); close up views of alkalinized Gifu B-129 (B) and MG-20 (C) plants. Plants at the two full developed leaves stage were watered with nutrient solution containing or lacking 10 mM NaHCO_3_ addition during 21 days.

Alkaline treatment caused a considerable reduction in stem elongation in both ecotypes, even though this effect was stronger in Gifu B-129 plants (Table 2, Figure 1 A). In turn, a detrimental effect on dry matter weight was also evident. In this case, MG-20 stem, leaf and root biomass was relatively more reduced with a total reduction of around 70%. In turn, alkalinity affected leaf and stem biomass of Gifu B-129 as well, whereas it had no effect on roots. As a consequence, the effect of treatments on total dry biomass was mild in this ecotype, ranging around 29%. Accordingly, Pn and WUE were negatively affected by the NaHCO_3_ addition in Gifu B-129, but not in MG-20 plants (Table 3). It should be noticed that at the last experimental stage, youngest leaves in alkalinized plants of Gifu B-129 were chlorotic (Figure 1 B), unlike those of alkalinized MG-20 plants which remained green (Figure 1 C). However, alkalinity decreased the total chlorophyll content in both ecotypes (Table 4). In addition, when stress treatment was extended to 2 months, some alkalinized Gifu B-129 plants died while MG-20 looked well adapted (results not shown). On these bases, we considered that Gifu B-129 was less tolerant to alkalinity than MG-20 plants.

**Table 2:** Stem length and dry weight in samples of *L. japonicus* MG-20 and Gifu B-129 plants.

	MG-20		Gifu B-129	
Growth parameter	Control	Alkaline	Control	Alkaline
Stem length (cm)	11.5±0.45 a	6.17±0.44 b	9.0±0.27 a	3.96±0.28 b
Root dry weight (g)	0.092±0.008 a	0.028±0.008 b	0.065±0.008 a	0.057±0.008 a
Stem dry weight (g)	0.032±0.003 a	0.012±0.003 b	0.019±0.002 a	0.008±0.002 b
Leaf dry weight (g)	0.12±0.032 a	0.032±0.010 b	0.047±0.004 a	0.028±0.004 b
Total dry weight (g)	0.24±0.02 a	0.07±0.02 b	0.13±0.012 a	0.093±0.013 b
Stem length reduction (%)		46.38±3.68 b		56.06±2.42 a
Total biomass reduction (%)		70.47±3.85 a		29.67±8.33 b

Seven-day-old plants were watered with 0.50 Hoagland’s nutrient solution, with or without the addition of 10 mM NaHCO_3_ during a period of 21 days. Results are the mean of 12 biological replicates ±SE. Statistical differences between control and treatments within each ecotype are shown as P<0.001 (Duncan’s post-hoc test).

**Table 3:** Gas exchange parameters transpiration rate (E), stomatal conductance (Gs), net photosynthesis (Pn) and water use efficiency (WUE) in basal and apical leaves of *L. japonicus* Gifu B-129 and MG-20 plants.

			E	Gs	Pn	WUE
			(mol H_2_O m^−2^ s^−1^)	(mmol H_2_O m^−2^ s^−1^)	(µmol CO_2_ m^−2^ s^−1^)	(µmol CO_2_ mmol^−1^H_2_O)
Basal leaf	Gifu B-129	control	2,22±0,25a	197±35a	3,6±0,6a	1,7±0,2a
		alkaline	1,48±0,29a	126±41a	1,5±0,6b	1,1±0,2b
	MG-20	control	2,18±0,25a	213±36a	3,1±0,6ab	1,5±0,2a
		alkaline	1,69±0,25a	178±36a	2,9±0,6ab	1,7±0,2a
Apical leaf	Gifu B-129	control	2,2±0,4a	197±41a	*2,9*±0,5a	1,28±0,25a
		alkaline	2,4±0,4a	214±41a	0,9±0,5b	0,43±0,25b
	MG-20	control	2±0,4a	200±41a	3,3±0,5a	1,63±0,25a
		alkaline	1,8±0,5a	193±47a	2,33±0,6ab	1,4±0,28a

Seven-day-old plants were watered with 0.50 Hoagland’s nutrient solution, with or without the addition of 10 mM NaHCO_3_ during a period of 21 days. Results are the mean of 12 biological replicates ±SE. Statistical differences between control and treatments within each ecotype are shown as P<0.001 (Duncan’s post-hoc test).

**Table 4:** Contents of chlorophyll a, b and total in apical leaves of *L. japonicus* Gifu B-129 and MG-20 plants.

		Chlorophyll a	Chlorophyll b	Total chlorophyll
Gifu B-129	control	0,9±0,1b	0,34±0,03a	1,24±0,14b
	alkaline	0,4±0,1c	0,18±0,03b	0,59±0,14c
MG-20	control	1,25±0,1a	0,25±0,43a	1,68±0,14a
	alkaline	0,82±0,1b	0,41±0,33a	1,15±0,14b

Seven-day-old plants were watered with 0.50 Hoagland’s nutrient solution, with or without the addition of 10 mM NaHCO_3_ during a period of 21 days. Results are the mean of 12 biological replicates ±SE. Statistical differences between control and treatments within each ecotype are shown as P<0.001 (Duncan’s post-hoc test).

It is well known that either Fe or Zn deficiencies may lead to leaf chlorosis [3]. Our results showed that there were no statistically significant change in the leaf Fe content between alkalinized and control plants, regardless the ecotype (Table 5). However, the leaf content of active Fe was diminished by NaHCO_3_ addition in Gifu B-129, but not in MG-20 plants (Table 6), suggesting that the leaf chlorosis observed in Gifu B-129 could be a consequence of such diminution. Alkalinity increased the total Fe concentration in the roots of Gifu B-129 plants and in roots and stems of MG-20 ones (Table 5). It also led to active Fe accumulation in roots and shoots of Gifu B-129 plants. By contrast, a notable reduction in Zn contents were found in both ecotypes, being this reduction more obvious in Gifu B-129 compared with in MG-20 (Table 5), indicating that Zn deficiency could also contribute to leaf chlorosis in Gifu B-129. Generally, plants exhibit Zn deficiency symptoms at shoot concentrations below a minimum of 15 to 20 ppm Zn/dry biomass. However this minimum has not been established for *L. japonicus* to date and more research is needed in this regard. On other hand, the higher alkalinity-induced Fe accumulation could be related to the observed reduction in Zn contents in both ecotypes, as Zn deficiency led to increased Fe accumulation in bean [32]. Interestingly, Fe accumulated in roots and stems (but not in leaves) of both *L. japonicus* ecotypes, what could be linked to the fact that Fe has shown to be immobilized in areas close to the vascular system [33].

**Table 5:** Total, leaf, stem and root iron and zinc contents in plants of *L. japonicus* MG-20 and Gifu B-129 plants.

Ion	Ecotype	Treatment	Total (ppm)	Leaf (ppm)	Stem (ppm)	Root (ppm)
Fe	MG-20	Control	1848,9±174,72b	179,44±22,97a	181,33±19,56a	1516±170,3a
	MG-20	Alkalinity	2681,54±174,72a	174,5±22,97a	306,7±55,46b	2199±170,3b
	Gifu B-129	Control	2065,23±165,76b	233,71±22,97a	306,65±18,76a	1579±161,6a
	Gifu B-129	Alkalinity	2609,01±198,12a	184,5±23,99a	354,73±35,12a	2091±193,1b
Zn	MG-20	Control	104,63±5,67a	27,64±1,69a	43,05±3,32a	36,42±3,38a
	MG-20	Alkalinity	39,16±5,4b	10,45±1,69b	7,02±4,34b	24,88±3,09a
	Gifu B-129	Control	122,49±5,4a	35,78±1,69a	64,91±3,46a	23,25±3,09a
	Gifu B-129	Alkalinity	33,27±5,4b	9,18±1,95b	14,28±4,34b	16,68±3,23b

Seven-day-old plants were watered with 0.5×Hoagland’s nutrient solution, with or without addition of 10 mM NaHCO_3_ over 21 days. Average data (±SE; n = 12) with the same letter within each ecotype are not significant different (Duncan, P<0.001).

**Table 6:** Available iron contents (nmol/gr de peso fresco) in leaves, stems and roots of *L. japonicus* Gifu B-129 and MG-20 plants.

		Root	Stem	Leaf
Gifu B-129	control	0,6±0,07b	0,53±0,08b	0,85±0,06a
	alkaline	1,18±0,07a	1,22±0,08a	0,51±0,06b
MG-20	control	0,51±0,07a	0,25±0,08a	0,59±0,06a
	alkaline	0,57±0,07a	0,41±0,08a	0,53±0,06a

Seven-day-old plants were watered with 0.5× Hoagland’s nutrient solution, with or without addition of 10 mM NaHCO_3_ over 21 days. Average data (±SE; n = 12) with the same letter within each ecotype×organ are not significant different (Duncan, P<0.001).

Fluorescence due to the presence of chlorophyll *a* in the PSII photosynthetic apparatus of plants has been proposed as a measure of the deleterious effects of different stresses such as temperature, drought, flooding, salinity, etc. [34]. Two photosynthesis-related indexes, the ratio of variable to maximal chlorophyll *a* fluorescence (*F*V/*F*M) and the performance index (PI_ABS_) have been shown to be particularly affected by diverse stresses in several plant species [35]. Low *F*V/*F*M values reflect a diminution in the ability of PS II to reduce the primary acceptor QA [36]. On the other hand, the PI_ABS_ is a multi-parametric expression taking into consideration the main functional steps of photosynthetic activity by a PSII reaction center complex [25]. Our results showed that both parameters were reduced by alkalinity in Gifu B-129 plants (Table 7). These data are in agreement with a recent report by Roosta [37] who informed a reduction in the FV/FM index of lettuce plants irrigated with an alkaline nutrient solution. In contrast, no variations in *F*V/*F*M and PI_ABS_ indexes were registered in MG-20 plants, reinforcing the notion of a higher alkalinity tolerance level in this ecotype compared with Gifu B-129. Also, it should be considered that alkalinized Gifu B-129 plants presented a high proportion of senescing leaves. Hence, as leaf senescence has been formerly shown to decrease the *F*V/*F*M ratio in cucumber [38], it is possible that senescence *per se* and not alkalinity could have provoked the decrease of the *F*V/*F*M ratio.

**Table 7:** Fv/Fm and PI_ABS_ measured on leaves of *L. japonicus* MG-20 and Gifu B-129 plants.

Ecotype	Treatment	Fv/Fm	PI abs
MG20	Control	0,84±0,01a	5,68±0,31 ab
MG20	Alkalinity	0,84±0,01a	6,15±0,31 a
Gifu	Control	0,83±0,01a	4,8±0,31 b
Gifu	Alkalinity	0,77±0,01b	1,22±0,31 c

Seven-day-old plants were watered with 0.5× Hoagland’s nutrient solution, with or without addition of 10 mM NaHCO_3_ over 21 days. Average data (±SE; n = 12) with the same letter within each ecotype are not significant different (Duncan, P<0.001).

### Microarray Analysis

#### Global transcription results

In order to gain insight into the transcriptomic changes involved in the generation of a new homeostatic cellular condition in alkalinized plants, we attempted to identify genes that were differentially expressed due to long-term alkaline treatment in both *L. japonicus* ecotypes. With this purpose, we performed an Affymetrix Lotus Genechip® analysis on pooled shoots and roots of plants from three independent experiments.

**Figure 2:**
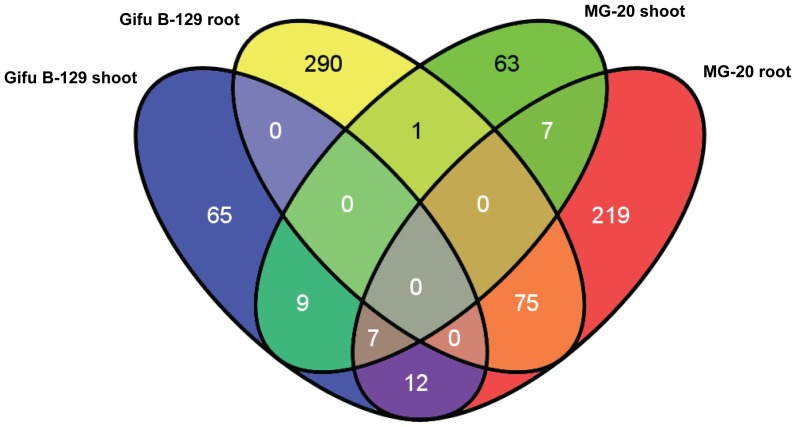
Venn diagram showing common and unique regulated genes by alkalinity between roots and shoots of MG-20 and Gifu B-129.

The analysis revealed quantitative and qualitative differences in transcriptional patterns between Gifu B-129 and MG20 ecotypes and also between shoots and roots within the same ecotype. Interestingly, only a minority of the probe sets elicited by alkalinity was shared between both ecotypes, and between roots and shoots of the same ecotype (Figure 2), indicating that the response was ecotype and organ-specific. Overall, 407 and 459 probe sets were regulated in MG-20 and Gifu B-129, respectively (P<0.05). The number of probe sets differentially expressed in roots was several-fold higher than that of shoots, regardless the ecotype, which is consistent with the role of these organs in detecting and triggering the main responses to cope with alkalinity.

On the base of shared putative function or common structural motifs, a functional annotation was assigned to 50% of the total probe sets using the MapMan software and the latest *L. japonicus* mapping file (Table 8). No annotation could be inferred for the remaining probe sets, which were regarded as genes with unknown function. In most of the functional categories where the rest of the genes were assigned, the number of up and down-regulated probe sets in each ecotype and organ combination was similar. An exception was found in some functional groups that were represented only by up-regulated probe sets. This is the case of glycolysis, gluconeogenesis and tricarboxylic acid cycle (TCA) functional categories. The number of probe sets in these categories and their expression level was higher in MG-20 than in Gifu B-129 plants, suggesting a higher potential of the first to supply energetic and carbon intermediates during the alkaline stress response. The most represented functional categories were miscellaneous (17,8%), transcription factors (13,5%), transport (11%), protein metabolism (10,3%), secondary metabolism (7,5%) and signalling (6,4%). In the following paragraphs, we describe the most relevant results within each of these groups (Table 8). MapMan illustrations depicting differentially expressed transcripts in plants confronted with NaHCO_3_ are provided as Figures S1–S4.

**Table 8:** Number of alkalinity-responsive probesets classified in each putative functional annotation, for each ecotype×organ combination.

Ecotype	Organ	Regulation	Amino acid metabolism	Biodegradation of Xenobiotics	Cell organisation	Cell wall	Development	DNA synthesis	Fermentation	Gluconeogenesis	Glycolysis	Hormone metabolism	Lipid metabolism	Major CHO degradation metabolism	Metal handling	Minor CHO metabolism	Miscelany	N-metabolism	Protein metabolism	Redox proteins	Secondary metabolism	Signalling	Stress	TCA	Transcription factors	Transporters	Unknown genes	Total 1	Total 2	Up/down-regulated genes
Gifu	Leaf	Down	0	1	0	1	0	0	1	0	0	1	0	0	3	0	2	0	0	0	0	1	0	0	1	0	6	17	93	4,5
		Up	0	0	2	0	0	0	0	0	0	1	0	1	2	0	1	0	8	1	1	3	2	0	12	16	26	76		
	Root	Down	3	0	1	5	4	4	0	0	0	6	5	2	0	1	20	0	7	2	6	4	7	0	14	12	38	141	382	1,7
		Up	2	0	2	4	4	1	1	0	1	12	4	1	4	3	34	0	24	1	23	17	15	1	21	8	58	241		
MG20	Leaf	Down	1	0	1	1	0	0	0	0	0	1	0	0	0	0	2	0	0	0	0	2	0	0	2	0	5	15	93	5,2
		Up	2	0	3	3	0	0	0	0	0	6	3	0	1	1	9	0	7	1	2	2	2	1	9	10	16	78		
	Root	Down	2	0	0	0	8	1	2	0	0	0	1	2	1	2	11	2	9	2	2	4	5	0	13	5	37	109	301	1,8
		Up	5	0	1	1	4	1	0	1	3	0	3	0	1	0	34	1	13	1	15	8	4	2	16	21	57	192		
Total			15	1	10	15	20	7	4	1	4	27	16	6	12	7	113	3	68	8	49	41	35	4	88	72	243	869	869	

Probesets with a mean absolute expression ratio of at least 1.5 (linear scale) and a *P*-value of P<0.05 in a *t*-test for significance were classified into the categories shown. Those probesets for which no annotation could be inferred were regarded as unknown.

#### Miscellaneous

Most of the probe sets in this group present homology to known Cytochrome P450 monooxygenase (CYP) genes (Table S1). CYP proteins plays critical roles in the synthesis of compounds involved in stress response, such as lignin, pigments, fatty acids, hormones and signalling molecules in all plant species [39]. The expression of CYP genes is tightly regulated by phytohormones during biotic and abiotic stresses [40]. Our analysis showed that CYP-like probe sets were regulated in both ecotypes, mainly in roots. However, the number of CYP regulated genes was higher in MG-20 than in Gifu B-129 plants. In addition, these genes show an up-regulated pattern in MG-20 in contrast to Gifu where down-regulation prevailed.

Another probe set included in this category correspond to a protein with a DHL domain which is exclusively regulated in MG-20 (2.9-Fold, Table S1). Several lines of evidence demonstrate that proteins from this family are usually involved in cell detoxification [41].

#### Transcription factors

Plant cells have evolved intricate signalling pathways to coordinate gene expression in response to external stimuli [42]. Among the genes involved in the transduction of these signals are transcription factors (TFs), which alter the expression of diverse stress-responsive genes. Many of the known TFs show differential regulation during abiotic stress in several important crop species [43], [44]. Our microarray analysis showed that several probe sets belonging to different TF families were regulated in roots and shoots of both ecotypes, such as bHLH, WRKY, GRAS, C2C2(Zn), NAC, MADS box, AP2/ERF, bZIP, G2-GARP. The number and diversity of these transcripts was considerably higher in Gifu B-129 than in MG-20. Moreover, the representatives from these categories in roots were more numerous compared to those in shoots (Table S1). These results suggested a higher stress perception in Gifu B-129, compared with MG-20, and confirmed the higher stress perception of roots. The top two TFs families with the largest number of genes differentially regulated were bHLH and MYB. Interestingly, we detected several bHLH-like probe sets strongly induced in MG-20 roots, whereas most of the bHLH-like genes were down-regulated or showed a very low induction in Gifu B-129 roots. In contrast, most MYB-like genes were up-regulated in roots of Gifu B-129, while no MYB-like genes were induced in MG-20. It has been suggested that the attenuated expression of a complex integrated by MYB and bHLH would allow metabolites from the flavonoid pathway to be diverted to the isoflavonoid biosynthesis in *L. japonicus* [45]. This is important since diverse roles for many of these secondary metabolites in plant defense against environmental and biotic stresses, as well as in the acclimatation to abiotic stress responses have been previously reported [46]. Members of some subgroups of these TF families were also shown to co-regulate the anthocyanin pathway in maize [47], or the proanthocyanidin pathway in *Arabidopsis* [48], *L. corniculatus* [49] and *L. japonicus* [50]. Based on above mentioned reports, it could be postulated that the observed divergence in the expression patterns of bHLH and MYB-like genes between Gifu B-129 and MG-20 might lead to the occurrence of distinct alkalinity-induced arrangements of flavonoids and isoflavonoids in each ecotype, which in turn might account for differences in plant tolerance between them. On the other hand, one of the most differentially regulated TFs was a G2-GARP-MYB-like transcript related to probe set chr1.tm0221.2_at, which was induced in shoots of both ecotypes but its expression level was higher in MG-20 than in Gifu B-129 (7,6-fold and 4-fold, respectively). Recently, a G2-GARP TF was shown to respond to arsenic in rice [51] and alkalinity in soybean leaf [7]. Therefore, it might be a common regulator of stress response in *L. japonicus*.

At last, putative HAP2 and AS2/LOB TFs were induced exclusively in Gifu B-129 roots. HAP2 TFs were shown to be involved in osmotic stress response in sorghum roots [52], whereas the AS2/LOB genes are involved in regulatory networks controlling root development in rice [53] and *Medicago truncatula* [54]. Interestingly, the addition of 10 mM NaHCO_3_ altered root architecture in the forage species *L. tenuis* [22]. On these grounds, we speculate that the regulation of these genes in this ecotype might be essential to change root morphology and trigger the plant response to alkalinization.

#### Transport

A group of five putative metal transporters were regulated in MG-20 roots and shoots, predominating the up- over down-regulation pattern (Table S1). The most up-regulated probe sets corresponded to genes coding for ZIP proteins, transporters involved in micronutrient homeostasis. It has been shown that these transporters are able to translocate various divalent cations, including Fe, Zn, Mn, and Cd [55]. The expression of ZIP genes is induced under Fe or Zn deficiency [55], which may come up as a result of high soil pH values [56]. Interestingly, no ZIP-like transporters were regulated in Gifu B-129 roots. Such a variation between both ecotypes in the expression pattern of this type of genes could be related to their different capability to cope with stress. However, further research addressing the ion specificity of these transporters is required in order to establish an unambiguous cause-effect relationship linking the expression of these genes with Fe and Zn accumulation in alkaline conditions.

On other hand, several probe sets related to transporters of highly diverse non-metal molecules were also regulated in both ecotypes. Among them, two ammonium transporters (AMT)-like transcripts were induced in roots, one in MG-20 (ljwgs_019948.1_at otra vez, mirar cual es el nombre completo; 2-fold), and the other in Gifu B-129 (ljwgs_019948.1_at lo mismo; 1.3-fold). Interestingly, the induction of the LeAMT1 gene in tomato roots was formerly observed under Fe-deficiency condition [57]. Also, three different putative nitrate transporters were up-regulated in MG20 roots, whereas another transcript of this type was down-regulated in roots of both ecotypes. The most highly induced of these transcripts in MG-20 (chr4.cm0046.61_at; 4,3-fold) is similar to an *Arabidopsis* nitrate transporter (AT1G59740) which functions in nitrate removal from xylem sap [58]. These results suggest a better nitrate assimilation in MG-20 compared to Gifu B-129, which is in line with the fact that Gifu B-129 plants experienced a higher level of active Fe-deprivation than MG-20 ones, since nitrate acquisition was shown to be limited under Fe deficiency in cucumber [59].

Another interesting observation is the down-regulation of three probe sets related to aquaporins (TIP) in Gifu B-129 roots. In turn, no regulation in members of this gene family was observed in MG-20 plants. It has been shown that intravesicular acidification inhibits the gating of plasma membrane aquaporins in storage root cells of *Beta vulgaris* [60]. However, nothing has been informed so far with regard to the effect of high pH on the expression of aquaporin genes in plants. We envision that forthcoming research will bring to light valuable new information on the effect of alkalinity on aquaporin functionality.

Several probe sets similar to *Arabidopsis* pleiotropic drug resistance (PDR) transporters (a type of ABC transporters) were strongly up-regulated in MG-20 and Gifu B-129 shoots, and more slightly induced in roots of both ecotypes. In plants, PDR-type transporters have been implicated in detoxification [61] and biotic defense response [62]. In addition, one probe set (ljwgs_125461.1_at) similar to a Phosphorous-glycoprotein (PGP, another type of ABC transporter) was exclusively regulated (3.1-fold change) in Gifu B-129 roots. Most of the plant PGPs characterized to the present have been implicated in auxin transport [63], although some evidence supports that AtPGP1 might function as ecto-phosphatase [64]. Since all probe sets putatively corresponding to auxin-responsive genes were downregulated in Gifu B-129 roots, it could be hypothesized that the observed induction of the PGP-like gene in this ecotype is a plant response to alkali-derived P deprivation.

#### Protein metabolism

Most of the regulated probe sets in this group correspond to genes involved in protein degradation. Interestingly, a higher proportion of up-regulated probe sets were observed in Gifu B-129, compared with MG-20 roots. It is well known that a primary function of protein degradation in stressed plants is to minimize the stress-induced damage by limiting abnormal protein accumulation and by supplying the amino acids necessary for maintaining cellular homeostasis and growth [65]. Thus, our microarray results suggest that a greater protein turnover could occur in Gifu B-129. The 2-isopropylmalate synthase (IPMS) is the first committed enzyme in leucine biosynthesis and a key part of essential aminoacids biosynthesis and primary metabolism [66]. In line with former results, we detected the induction of four transcripts similar to IPMS genes, exclusively in MG-20 roots, suggesting a more active aminoacids biosynthesis in this ecotype.

Several expression studies indicated the possible involvement of plant matrix metalloprotease (MMPs) in the response to abiotic stresses, such as salinity [67], wounding, dehydration [68], and low temperature [69]. Accordingly, we detected a regulated probe set (chr2.cm1150.57_at) similar to At1g70170, a matrix metalloprotease (MMP) with funtions in zinc ion binding and proteolysis, which was 3-fold induced in Gifu B-129 roots. In contrast, another MMP-like probe set (chr2.cm1150.58_at) detected in MG-20 roots was downregulated.

#### Metal handling

Ferric chelate reductase (FRO) enzymes have been shown to reduce and solubilise Fe(III) chelates at the root surface in *Arabidopsis* [70]. On other hand, nicotianamine synthase (NAS) is a key enzyme in the synthesis of nicotianamine (NA) in non-graminaceous plants, which do not produce phytosiderophores to acquire Fe. In these plants, NA chelates metal cations including those formed by Fe, suggesting a function in the internal mobilization of Fe and other metals [3], [71]. According to our microarray results, several FRO-like genes were downregulated in Gifu B-129 shoots but induced in roots of both ecotypes (showing a higher regulation on MG-20). In parallel, two nicotianamine synthase-like genes were induced in shoots upon alkalinization. Whereas one of them was exclusively regulated in Gifu B-129 (chr1.cm0206.26_at), the second probe set (chr6.cm0539.8_at) was regulated in both ecotypes, being its expression level higher in MG-20 than in Gifu B-129. Taken together, the transcriptomic pattern of FRO, NAS and ZIP-like genes displayed in our microarray suggests that under the alkaline condition, MG-20 would have a higher ability to mobilize Fe in leaves and to reduce ferric chelates in the root surface than Gifu B-129. This is in line with our results showing a higher alkalinity-induced total Fe accumulation in MG-20, compared with Gifu. In turn, the occurrence of a more suitable machinery for Fe acquisition in MG-20 may help to explain the better performance of this ecotype under the alkaline condition.

It has been shown that after Fe is loaded into the root xylem from the pericycle, a ferric reductase defective (FRD) gene coding for a multidrug and toxin efflux protein, facilitates Fe chelation to citrate and their subsequent transport from roots to shoots [72]. Our microarray data also showed the induction of two FRD-like probe sets, one in MG-20 shoots and the other in Gifu B-129 roots. Possibly, the asymmetric location of alkalinity-regulated FDR-like genes between MG-20 and Gifu B-129 could contribute to explain the differences in the levels of shoot Fe accumulation found between both genotypes.

#### Secondary and hormone metabolism

Most of the differentially expressed probe sets related to secondary metabolism were regulated in roots, where induction prevailed over down-regulation. In both ecotypes, the majority of induced probe sets were similar to genes that participate in flavonoids synthesis. As stated above, these compounds play a role in plant defence against environmental and biotic stresses. In addition, two probe sets assignable to the lignin biosynthetic enzymes cinnamyl alcohol dehydrogenase (CAD) were induced in roots and shoots of MG-20 plants, whereas no gene of this type was regulated in Gifu.

Several hormone-related probe sets were differentially expressed in MG-20 and Gifu B-129 upon alkalinization. Those that were regulated in shoots were mostly induced and showed homology with auxin-responsive genes, as well as genes involved in gibberellin and jasmonic acid biosynthesis. Suggestively, no regulation of probe sets putatively involved in hormone biosynthesis was detected in MG-20 roots. Moreover, a probe set (cm0584.17_at) similar to a negative regulator of the gibberellin signal transduction pathway (RGA) [73] was induced in roots of this ecotype. In contrast, several probe sets putatively related to ABA and ethylene biosynthesis were induced in Gifu B-129 roots, where the down-regulation of genes probably involved in auxin and cytokinin response or biosynthesis was also observed. In *Arabidopsis*, the expression of the senescence-related *AtMYBL* TF was induced by ABA [74]. In turn, the overexpression of *AtMYBL* increased the levels of the *Senescence-related gene 1* (*SRG1*), which was reported to increase in senescent organs and hence, it was proposed as a marker for senescence in that model plant [75]. The fact that three SRG-like probe sets (chr2.cm0124.31_at, ljwgs_067741.2_at and ljwgs_084207.1_at), along with four MYB-like TF were induced in Gifu B-129 (whereas these types of genes were down-regulated or undetected in MG-20) is in line with the higher root turnover of Gifu roots suggested above, and congruent with the view that these roots were senescing, unlike those of MG-20.

#### Stress perception and signaling

Plant stress perception is followed by the expression of a large number of genes involved in morphological or physiological processes that increase survival under stressful condition. This process relays on a number of cell surface proteins, including calcium-binding proteins, receptors-like kinases (RLKs), G-protein-coupled receptors and two-component histidine kinase receptors [42]. Our results showed the regulation of 41 probe sets putatively representing several of these stress receptors. These types of genes were more abundant in roots than in shoots, in congruence with the fact that roots are in direct contact with the stress source.

Some types of G-proteins called GTP-binding proteins participate of vesicle trafficking and have a role in plant adaptation to stress and damage repair [76]. Interestingly, a G-protein-like probe set (gi1370149_at), similar to the GTP- binding protein AT1G06400 was 2.2-fold induced in Gifu B-129 shoots, whereas no GTP-binding-like gene was regulated in MG-20, indicating a possible higher requirement for damage repair in the former ecotype.

#### Other genes of interest

Among all regulated transcripts in our microarray, the most up-regulated was chr1.cm0109.32_at, which was induced in MG-20 and Gifu B-129 shoots (7.7 and 4.2-fold, respectively). This transcript showed homology to the Phloem protein ATPP2-B15 of *Arabidopsis*. PPs proteins are poly-GlcNAc-binding lectins [77], whose expression is developmentally related to defined stages of phloem differentiation [78]. PP2 also interacts with mesophyll plasmodesmata to increase the size exclusion limit and traffic cell-to-cell in *Arabidopsis* [79]. As this transcript was more highly induced in MG-20 than Gifu B-129 shoots, it is suggested that the former ecotype has greater sugar transportation ability.

The carbonic anhydrase (CA) enzyme catalyzes the rapid interconversion of bicarbonate and protons to dioxide and water (or vice versa), by removing a water molecule from carbonic acid. In plants, CA is involved in diverse biological processes including pH regulation, respiration and photosynthesis [80]. In addition, Zn deficiency induces a decrease in the activity of CA [81], which in turn has been proposed as an indicator for diagnosing Zn deficiency in plants [82]. Our microarray results revealed the regulation of three CA-like genes. In this trend, ljwgs_021122.2_at and chr2.cm0201.34_at were 5 and 4.3-fold induced in MG-20 roots, respectively, whereas another probe set of this type (ljwgs_048469.1_at) was induced in Gifu B-129 roots, even though showing a lower expression level (1.3-fold). These results reinforce the view that roots of the second ecotype could have experienced Zn deficiency, unlike those of MG-20.

Another highly regulated probe set (chr5.CM0456.15_at), a putative acidic endochitinase (CHIB1), was 4-fold induced in MG-20 shoots. Interestingly, this transcript was formerly shown to be strongly up-regulated (5.1-fold) in shoots of this ecotype when challenged with *Pseudomonas syringae* pv. tomato [83], suggesting that it could be involved in a generalized mechanism of plant response to biotic and abiotic stresses.

The main cause of declination in plant growth and productivity under stress conditions is the oxidative damage at the cellular level [84]. Plant thioredoxins are thought to act as regulators of scavenging mechanisms and as components of signalling pathways in the plant antioxidant network [85]. Their induction was higher in MG-20 (1.7 vs 1-fold), suggesting a greater antioxidant potential in this ecotype. Finally, several peroxidases-like genes were also slightly regulated in root of both genotypes, but the relation of down-regulated to induced transcripts was similar in both ecotypes.

### Validation of Microarray Data by Real-time Quantitative PCR

**Figure 3:**
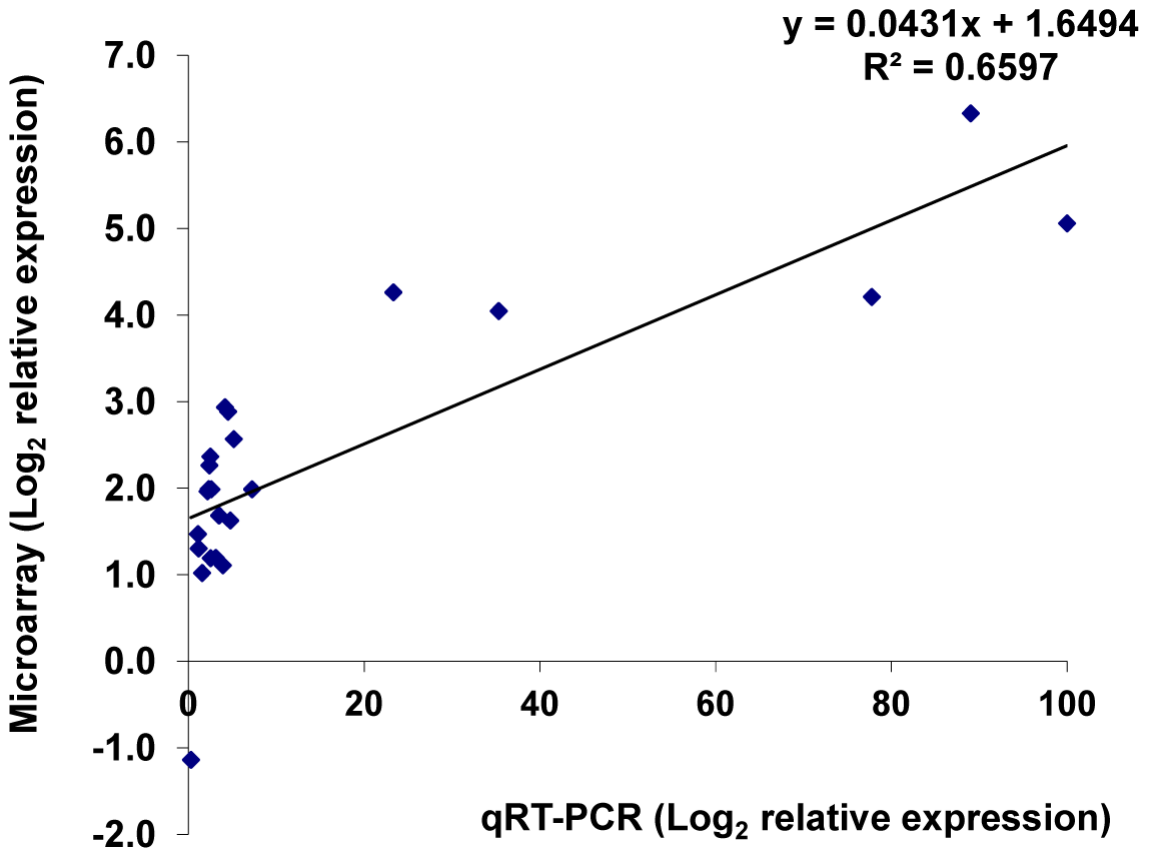
Comparison of microarray and quantitative real-time PCR data for 15 selected genes. Symbols represent Log_2_ transformation of mean expression levels relative to control treatments.

To validate microarray results presented in this study, we analyzed the expression data of 20 randomly chosen genes by qRT-PCR (Table 9), using cDNA from three independent biological replicates. A linear regression analysis of microarray and qRT-PCR values (Figure 3), yielded a R^2^ = 0.6597 (Pearson’s correlation r = 0.86). In addition, although the magnitude of the transcript abundance varied, the gene expression patterns obtained by microarray and qRT-PCR were similar.

**Table 9:** Microarray and qRT-PCR analysis of expression of 20 randomnly selected genes in *Lotus japonicus* roots and leaves treated with NaHCO_3_ during 21 days.

Probeset	Relative expression (fold change)		Ecotype×organ
	Microarray	qRT-PCR	
chr1.cm0800.52	1	1,552	Gifu B-129 root
ljwgs_021886.2	1,109	3,918	MG-20 leaf
ljwgs_063085.1	1,192	2,526	MG-20 leaf
chr1.cm0378.1	1,195	3,109	MG-20 leaf
cm0528.2	1,304	1,156	MG-20 root
cm0528.2	1,627	4,757	MG-20 leaf
cm0528.2	1,685	3,477	Gifu B-129 leaf
chr3.cm0279.2	1,987	2,607	MG-20 root
ljwgs_147904.1.1	1,99	2,301	Gifu B-129 root
ljwgs_055792.1	2	2,168	MG-20 root
chr1.cm0433.8	2,264	2,378	Gifu B-129 root
chr5.cm0019.23	2,365	2,492	MG-20 root
chr6.cm0437.7	2,567	5,155	MG-20 leaf
ljwgs_038566.1	2,884	4,512	MG-20 root
ljwgs_049882.1	2,935	4,155	MG-20 root
chr5.cm0456.170.r2.d	4,047	35,302	MG-20 leaf
ljwgs_086126.1	4,209	77,753	MG-20 leaf
Ljwgs_012445.1	4,262	23,316	MG-20 leaf
ljwgs_124992.1	5,059	99,971	MG-20 leaf
ljwgs_011581.2	6,33	89,008	MG-20 leaf

## Conclusions

We report in this work a comparative analysis of alkali-induced changes on growth, physiological parameters and gene expression in the *L. japonicus* ecotypes MG-20 and Gifu B-129. Plant growth assessment revealed substantial differences in tolerance to soil alkalinity between both ecotypes, which were reflected by physiological analysis and metal accumulation measurements. These results support the notion that overall, MG-20 plants displayed a higher tolerance level to alkaline stress than Gifu B-129.

Importantly, plants were exposed to alkalinity for 21 days, a period much longer than those used by other authors in similar works. This approach allowed the detection of genes involved in metabolic pathways shown to be activated by nutrient deficiency in other plant species, in particular Fe and Zn. Such results suggest that MG-20 and Gifu B-129 ecotypes differed in their regulation of genes commonly believed to be involved in the generation of a new Fe/Zn homeostatic cellular condition. Thus, MG-20 displayed higher number and expression level of several metal transporters (Fe, Zn and Cd), such as ZIP, NAS and PDR-like transcripts, as well as transcripts coding for proteins shown to reduce Fe (III) chelates at the root surface to form soluble Fe (FRO). Taken together, the transcriptomic pattern suggests that under alkaline conditions, MG-20 has a more suitable machinery for Fe acquisition and for Fe and Zn transport than Gifu B-129.

On the other hand, the MG-20 ecotype showed a greater number of regulated transcripts (and with higher expression levels) putatively involved in glycolysis, gluconeogenesis and TCA. This suggests that this ecotype could count with a better supply of energetic and carbon intermediates during alkaline stress. In this regard, the induction of a transcript putatively involved in phloem differentiation (PP2) in MG-20, but not in Gifu B-129, is in line with these results and indicates a greater sugar transportation ability in the first ecotype. The last results agrees also with the fact that CYP-likes genes (a gene family playing critical roles in the synthesis of plant compounds involved in stress response) were induced in a higher number and expression level in MG-20 than in Gifu B-129. As a whole, this information advocates for a superior primary and secondary metabolism activity in MG-20, compared with Gifu B-129, including the biosynthesis of plant defense-related compounds. On the other hand, a bulk of genes that were exclusively, or more regulated in Gifu B-129 than in MG-20, was indicative of a greater stress level in the first ecotype. Among these genes, we detected up-regulated MMP with functions in Zn ion binding/proteolysis and a higher proportion of up-regulated to down-regulated probe sets putatively involved in protein-degradation, suggesting a higher protein turnover in the first ecotype. In this trend, we also identified the induction in Gifu B-129 shoots of a probe set similar to a G-protein, with role in plant adaptation to stress and damage repair and the induction of senescence-related genes in roots. On other hand, differences between both ecotypes in the expression patterns of bHLH and MYB-like genes constitute a hint that both ecotypes could display distinct arrangements of flavonoid and isoflavonoid compounds, which in turn could account for a lower or higher plant tolerance to biotic and abiotic stresses.

In the present work, we showed that the Gifu B-129 and MG-20 ecotypes differ in their homeostatic response to long-term alkalinity and provided a set of selected, differentially expressed genes deserving further investigation.

Finally, our results, in addition to a previous study showing contrasting responses to a hemibiotrophic bacteria [83], tempt us to propose Gifu B-129 and MG-20 ecotypes as useful models to search for common and distinct tolerance mechanisms to biotic and abiotic stress responses.

## Supporting Information

Figure S1 **MapMan illustration depicting transcripts from the “Cell functions overview” bin regulated in MG-20 leaves, upon alkalinization.** Transcriptomic data from NaHCO_3_-treated plants was compared to respective untreated controls. Genes that were shown to be differentially expressed were mapped using the MapMan software (http://mapman.gabipd.org). Log fold change ratios are indicated as a gradient of red (down-regulated) and blue (up-regulated).
(TIF)

Figure S2 **MapMan illustration depicting transcripts from the “Cell functions overview” bin regulated in MG-20 roots, upon alkalinization.** Transcriptomic data from NaHCO_3_-treated plants was compared to respective untreated controls. Genes that were shown to be differentially expressed were mapped using the MapMan software (http://mapman.gabipd.org). Log fold change ratios are indicated as a gradient of red (down-regulated) and blue (up-regulated).
(TIF)

Figure S3 **MapMan illustration depicting transcripts from the “Cell functions overview” bin regulated in Gifu B-129 leaves, upon alkalinization.** Transcriptomic data from NaHCO_3_-treated plants was compared to respective untreated controls. Genes that were shown to be differentially expressed were mapped using the MapMan software (http://mapman.gabipd.org). Log fold change ratios are indicated as a gradient of red (down-regulated) and blue (up-regulated).
(TIF)

Figure S4 **MapMan illustration depicting transcripts from the “Cell functions overview” bin regulated in Gifu B-129 roots, upon alkalinization.** Transcriptomic data from NaHCO_3_-treated plants was compared to respective untreated controls. Genes that were shown to be differentially expressed were mapped using the MapMan software (http://mapman.gabipd.org). Log fold change ratios are indicated as a gradient of red (down-regulated) and blue (up-regulated).
(TIF)

Table S1 **List of main functional categories and probesets regulated in response to alkalinity, mentioned in Results and Discussion.**
(XLS)
