# Supplementary figures and images for: Response to Long-Term NaHCO3-Derived Alkalinity in Model Lotus japonicus Ecotypes Gifu B-129 and Miyakojima MG-20: Transcriptomic Profiling and Physiological Characterization
Source: PLoS One. 2014 May 16;9(5):e97106. doi: 10.1371/journal.pone.0097106 (PMC4024010; doi:10.1371/journal.pone.0097106)

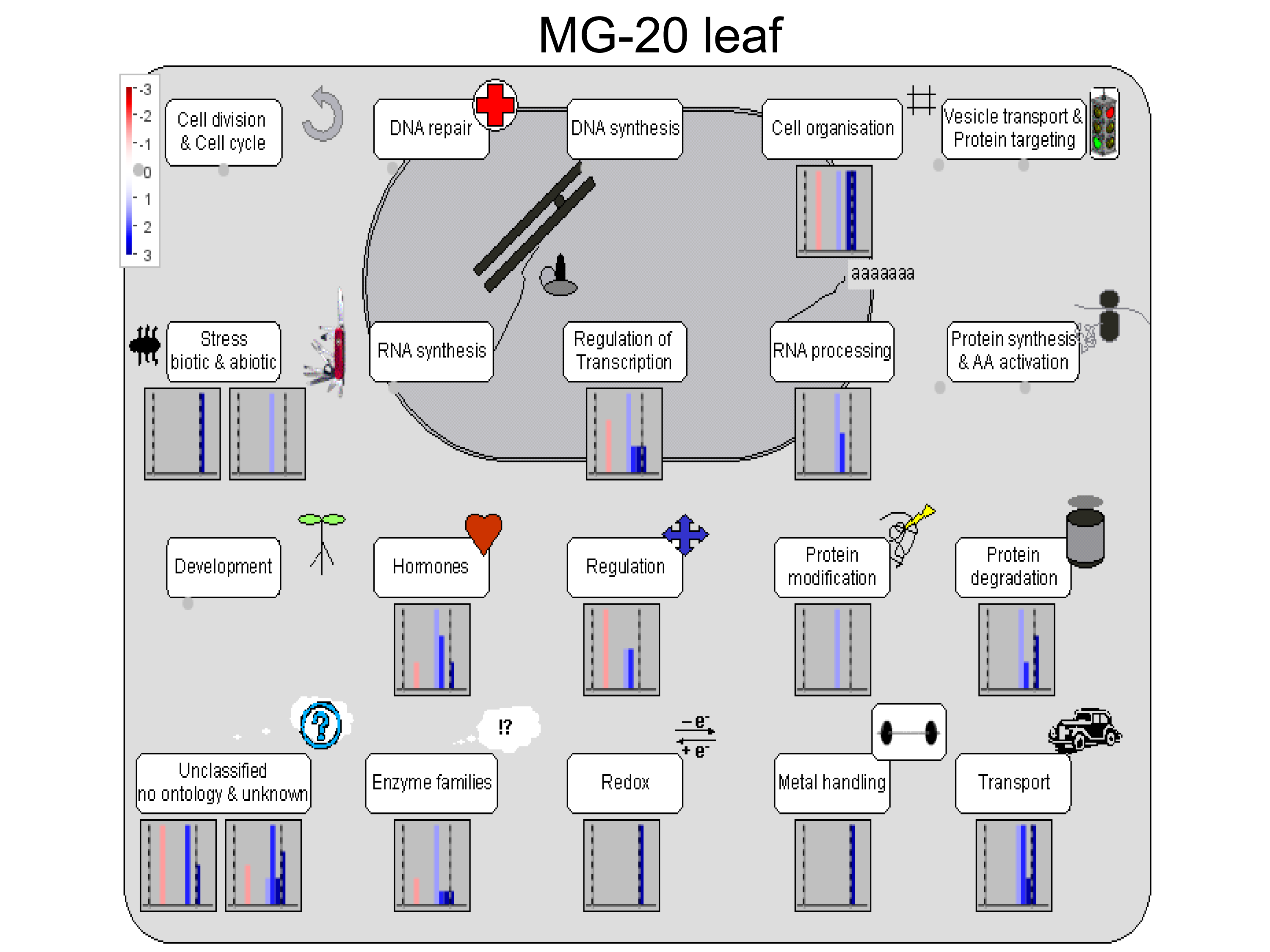

Supplement: Figure S1 — MapMan illustration depicting transcripts from the “Cell functions overview” bin regulated in MG-20 leaves, upon alkalinization. Transcriptomic data from NaHCO3-treated plants was compared to respective untreated controls. Genes that were shown to be differentially expressed were mapped using the MapMan software (http://mapman.gabipd.org). Log fold change ratios are indicated as a gradient of red (down-regulated) and blue (up-regulated). (TIF) [file pone.0097106.s001.tif]

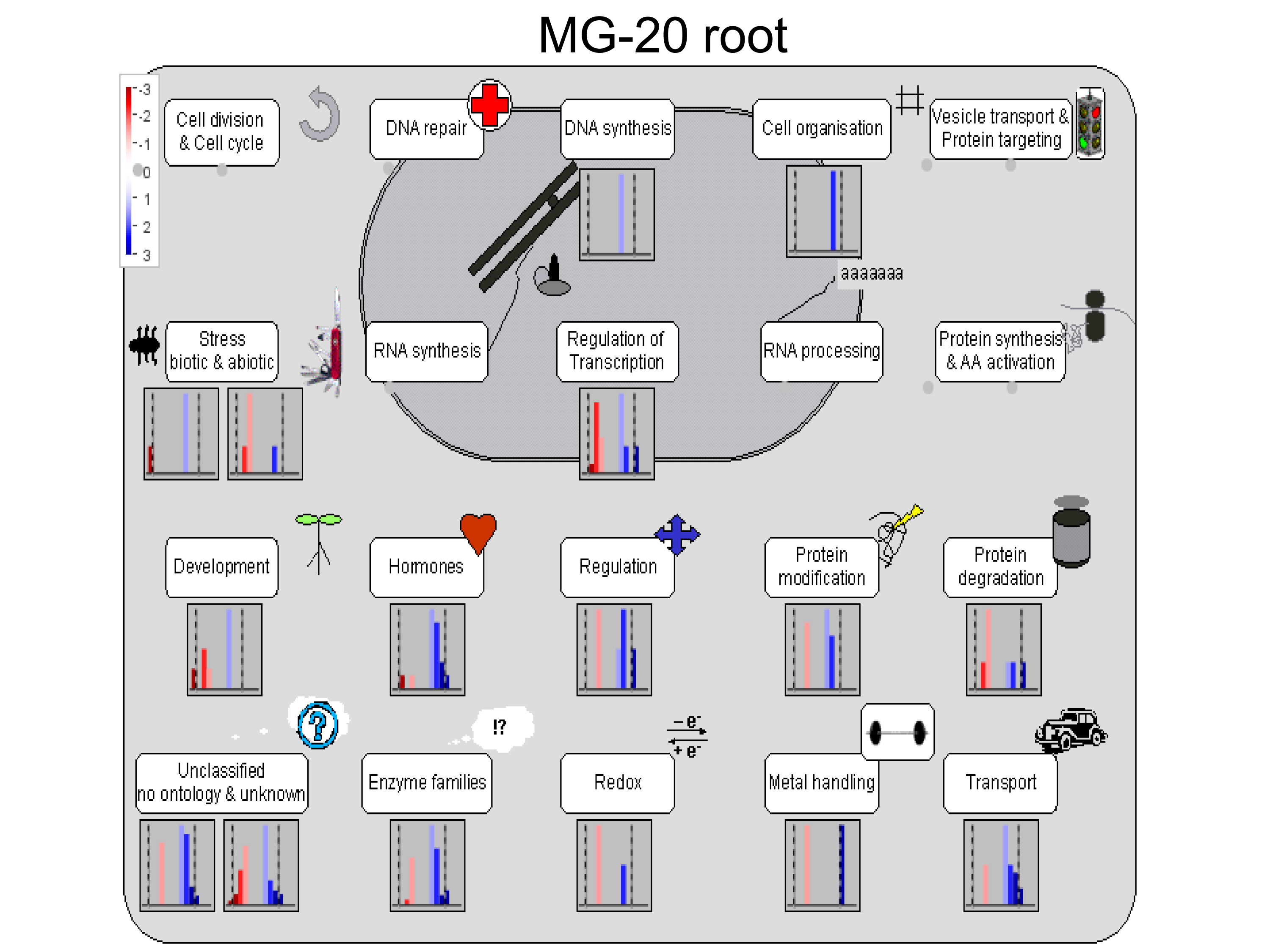

Supplement: Figure S2 — MapMan illustration depicting transcripts from the “Cell functions overview” bin regulated in MG-20 roots, upon alkalinization. Transcriptomic data from NaHCO3-treated plants was compared to respective untreated controls. Genes that were shown to be differentially expressed were mapped using the MapMan software (http://mapman.gabipd.org). Log fold change ratios are indicated as a gradient of red (down-regulated) and blue (up-regulated). (TIF) [file pone.0097106.s002.tif]

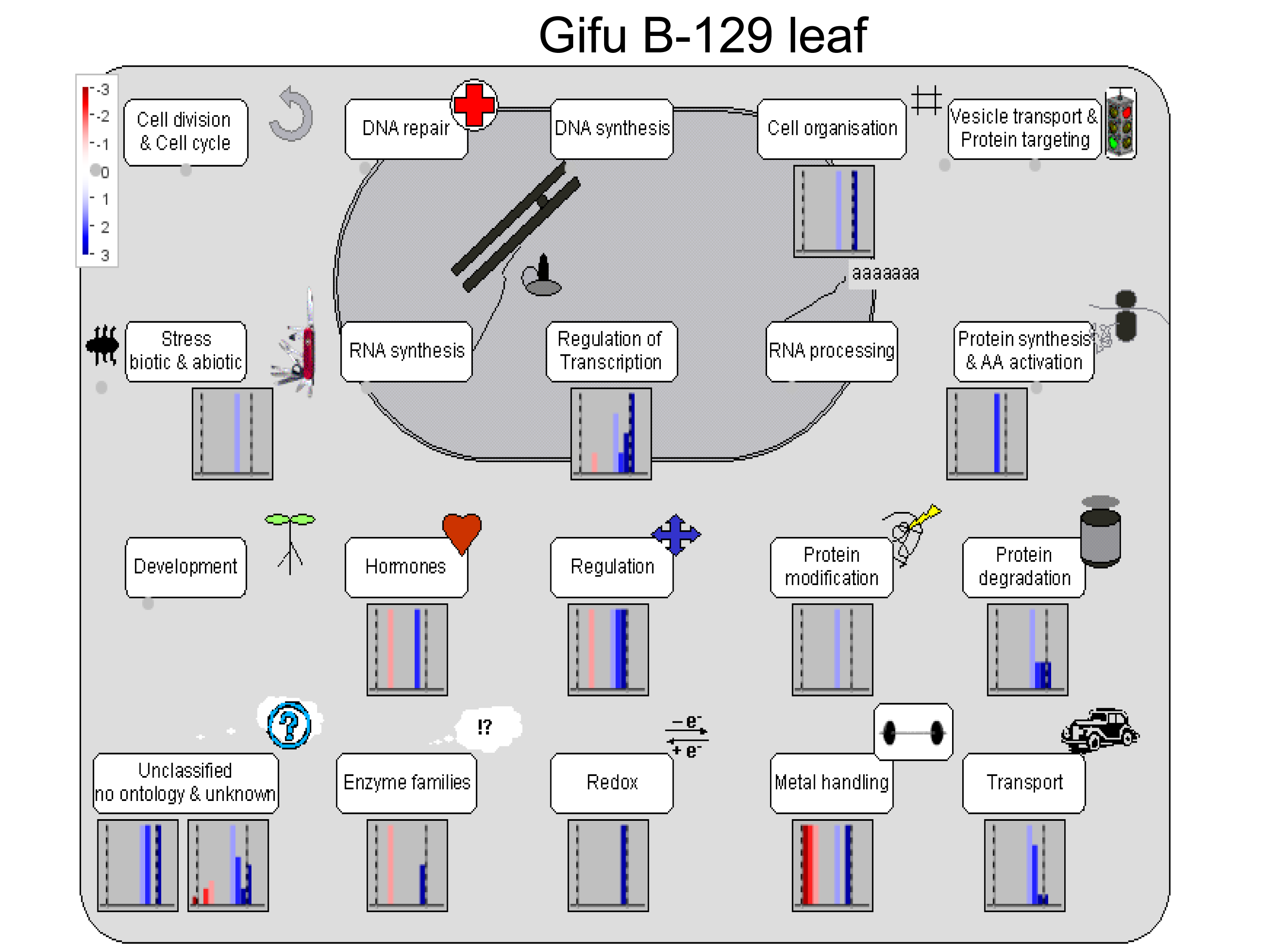

Supplement: Figure S3 — MapMan illustration depicting transcripts from the “Cell functions overview” bin regulated in Gifu B-129 leaves, upon alkalinization. Transcriptomic data from NaHCO3-treated plants was compared to respective untreated controls. Genes that were shown to be differentially expressed were mapped using the MapMan software (http://mapman.gabipd.org). Log fold change ratios are indicated as a gradient of red (down-regulated) and blue (up-regulated). (TIF) [file pone.0097106.s003.tif]

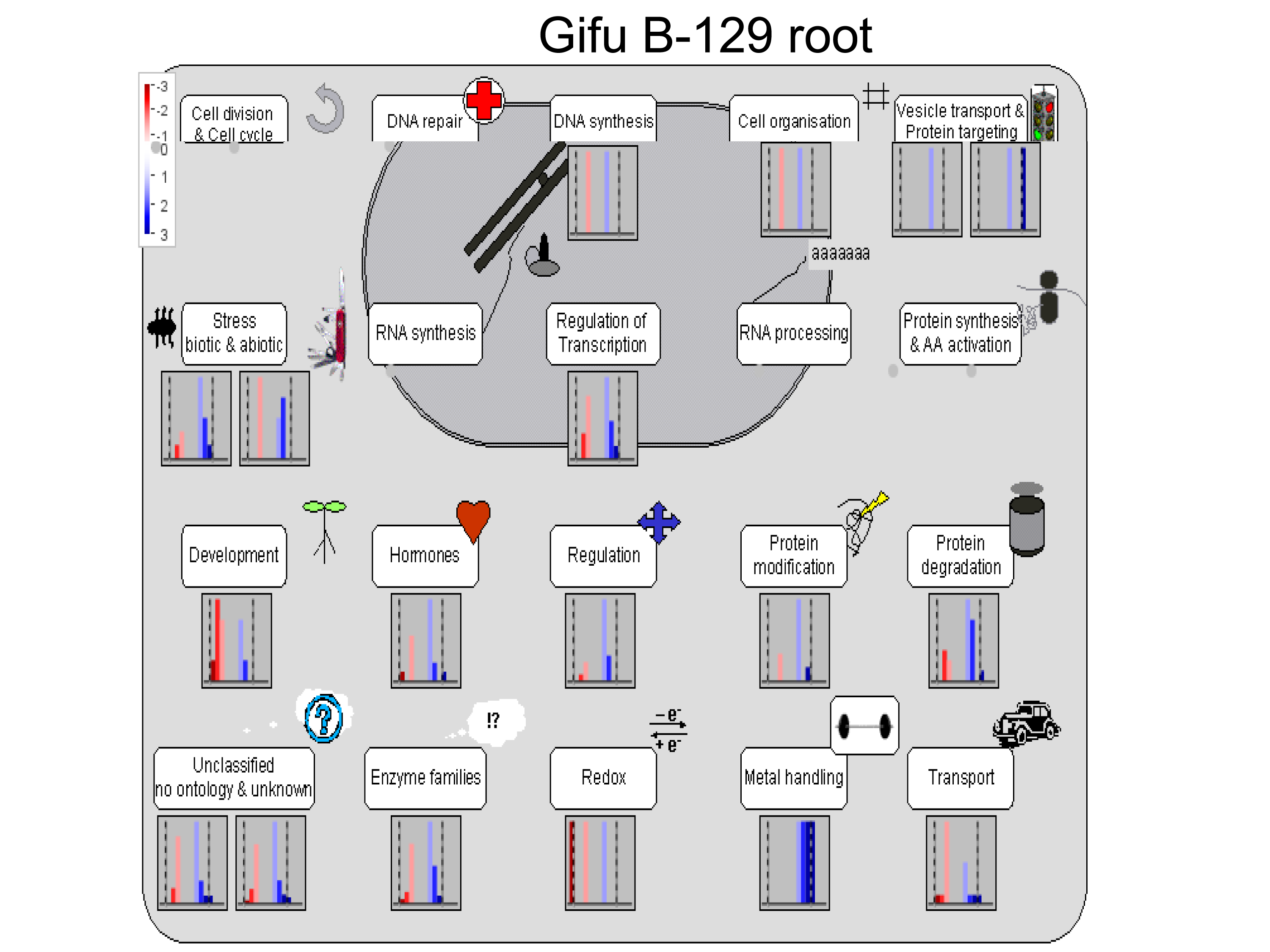

Supplement: Figure S4 — MapMan illustration depicting transcripts from the “Cell functions overview” bin regulated in Gifu B-129 roots, upon alkalinization. Transcriptomic data from NaHCO3-treated plants was compared to respective untreated controls. Genes that were shown to be differentially expressed were mapped using the MapMan software (http://mapman.gabipd.org). Log fold change ratios are indicated as a gradient of red (down-regulated) and blue (up-regulated). (TIF) [file pone.0097106.s004.tif]
